# Lifestyle behaviour changes associated with osteoarthritis: a prospective cohort study

**DOI:** 10.1038/s41598-024-54810-6

**Published:** 2024-03-14

**Authors:** Norman Ng, Lynne Parkinson, Wendy J. Brown, Rachael Moorin, G. M. E. E. Geeske Peeters

**Affiliations:** 1 School of Human Movement and Nutrition Science (#26B), The University of Queensland, Blair Drive, St Lucia Campus, Brisbane, QLD 4072 Australia; 2 University of Newcastle, Newcastle, Australia; 3 Faculty of Health Sciences and Medicine, Bond University, Gold Coast, Australia; 4 School of Public Health, Curtin University, Perth, Australia; 5 Department of Geriatric Medicine, Radboud University Medical Centre, Nijmegen, Netherlands

**Keywords:** Health behaviour, Sitting, Exercise, Arthritis, Cohort studies, Women’s health, Osteoarthritis, Lifestyle modification

## Abstract

The aim of this prospective cohort study was to compare changes in lifestyle behaviours over nine years in women who were and were not diagnosed with osteoarthritis (OA). Data were from the 1945–51 cohort of the Australian Longitudinal Study on Women’s Health (aged 50–55 in 2001) who completed written surveys in 2001, 2004, 2007 and 2010. The sample included 610 women who were, and 3810 women who were not diagnosed with OA between 2004 and 2007. Descriptive statistics were used to assess changes in lifestyle behaviours (weight, sitting time, physical activity, alcohol and smoking) in the two groups, over three survey intervals: from 2001–2004 (prior to diagnosis); from 2004–2007 (around diagnosis); and from 2007–2010 (following diagnosis). Compared with women without OA (28%), a greater proportion of women with OA (38%) made at least one positive lifestyle change (p < 0.001). These included losing > 5 kg (9.8% vs. 14.4%, p < 0.001), and reducing sitting time by an hour (29.5% vs. 39.1%, p < 0.001) following diagnosis. However, women with OA also made negative lifestyle changes (35% vs. 29%, p < 0.001), for example, gaining > 5 kg around the time of diagnosis (21.4% vs. 14.5%, p < 0.001) and increasing sitting time by an hour following diagnosis (38.4% vs. 32.3%, p = 0.003). More women with OA also started smoking following diagnosis (8.9% vs. 0.8%, p < 0.001). While some women made positive changes in lifestyle behaviours during and following OA diagnosis, others made negative changes. Consistent support from clinicians for managing OA symptoms may enable patients to make more positive changes in lifestyle behaviours.

## Introduction

Osteoarthritis (OA) is one of the most common forms of musculoskeletal disorder, characterised by pain, stiffness, swelling and limited joint movement^1,2^. The disease is a leading cause of pain and disability in most Western developed countries^3–5^. In 2017–18, 2.2 million Australians were estimated to have OA, which affects twice as many women as men (12% vs 6.8%). Direct health expenditure of AUD 3.9 billion was attributed to OA between 2019 and 2020^6,7^.

Over the last decade, evidence-based recommendations have been made for the management of hip and knee OA. These include both non-pharmacological and pharmacological therapies, with surgical options available for those who fail to respond to these therapies^8,9^. The goals for management are to reduce joint pain and stiffness, maintain and improve joint mobility, improve muscle strength, limit subsequent joint damage, reduce activity limitations and improve health-related quality of life. Given the cost-effectiveness and safety of lifestyle changes in managing OA, recommendations include exercise and weight loss as core components of an effective management plan^10^. Several studies have reported reduced knee pain and improved physical function and quality of life from land-based and aquatic exercises^11,12^. Evidence suggests a link between OA and metabolic syndrome, which further warrants the need for positive lifestyle behavioural management strategies as effective non-invasive therapies^13^. However, little is known about the uptake of positive and negative lifestyle behaviours by women in the community after the onset of the disease. Knowing which lifestyle behaviours are and are not commonly adopted is important, because this information could inform the development of future intervention strategies.

The aim of this study was to compare changes in lifestyle behaviours over 9-years in women with and without OA, in the period prior to OA diagnosis, around the time of the OA diagnosis, and in the period following the OA diagnosis.

## Methods

The Australian Longitudinal Study on Women’s Health (ALSWH) is an ongoing population-based study of factors affecting the health and well-being of three cohorts of women born in 1975–81, 1946–51 and 1926–1931. Recruitment, data collection procedures and attrition have been described in more detail elsewhere^14^. In summary, in 1996, women were selected randomly from the national Medicare health insurance databased, which includes all citizens and permanent residents of Australia. The sample was reasonably representative of the general population of Australian women, although there was overrepresentation of Australia-born and university-educated women. All methods and experimental protocols were approved by the Universities of Newcastle and Queensland and all participants provided informed consent [ALSWH Protocol # H-2011-0371 (2012/HE000132)]. All methods were performed in accordance with the relevant guidelines and regulations. Detailed methods are available from www.alswh.org.au.

For the current study, we adopted a nested case–control design. Data were from the 2001, 2004, 2007 and 2010 surveys of the 1946–51 cohort who completed the first survey in 1996 (N = 13,716), with follow-up surveys every three years since 1998. These analyses included data from 4420 women, of whom 610 first reported having been diagnosed with OA in 2007 (when aged 56–61 years,) and a comparison sample of 3810 women in the same cohort who did not report any form of arthritis between 2001 and 2010. Data from surveys prior to 2001 were not included, as questions about OA were not included in prior surveys. The sample comprised women living in Australia, whose health care would involve mostly doctors, allied health professionals, and self-management.

In the 2007 survey, women were asked: “In the past three years have you been diagnosed or treated for”: a) osteoarthritis; b) rheumatoid arthritis; c) other arthritis. Women could report having more than one type of arthritis. Results from a systematic review showed that self-report of rheumatoid (sensitivity = 0.88, specificity = 0.93) and osteoarthritis (sensitivity = 0.75, specificity = 0.89) is acceptable for use in large-scale studies in which rheumatologist examination is not feasible^15^. Moreover, asking about doctor diagnosis increased the likelihood of capturing a sample that received lifestyle advice on diagnosis, commensurate with guidelines. The specific type of lifestyle advice provided at diagnosis was not collected in the surveys. Two groups were defined; ‘OA group’ and ‘No OA group’. The OA group reported ‘OA’ or ‘other arthritis’ (i.e., not rheumatoid arthritis) in 2007; they did not report arthritis of any type in 2001 or 2004, and did not report use of “arthritis medicines” at any survey before 2007 or use disease-modifying anti-rheumatic drugs (DMARDs) at any time. The No OA group did not report arthritis of any kind in 2001, 2004, 2007 or 2010; they did not report use of medications for arthritis at any survey and did not use DMARDs at any time.

Socio-demographic (age, education, marital status, area of residence) and health variables were taken from the 2001 survey and categorised as shown in Table 1. Sitting time was based on responses to this question: “How many hours each day do you typically spend sitting down while doing things like visiting friends, driving, reading, watching television, or working at a desk or computer?”^16^. Hours spent sitting on a weekday and a weekend day were averaged ([week day*5 + weekend day*2]/7) to estimate the mean sitting time in hours per day. Physical activity (PA) was measured using questions based on the Active Australia survey^17^. Self-reported frequency and duration of walking briskly, moderate and vigorous leisure-time activities during the last week were multiplied by MET scores that reflect the average intensity of the activities in that category: walking briskly = frequency*duration*3.0; moderate activities = frequency*duration*4.0; vigorous activities = frequency*duration*7.5. A MET is defined as 1 kcal/kg/hour or 3.5 ml/kg/min, which is equivalent to the energy cost or oxygen uptake of sitting quietly^18^.

**Table 1:** Socio-demographic, lifestyle and health-related characteristics of women who were and were not diagnosed with OA between 2004 and 2007. All data are from the 2001 survey^a^ (N = 4420).

	Women diagnosed with OA (n = 610)	Women not diagnosed with OA (n = 3810)	p-value^b^
Age y Mean (SD)	52.40 (1.49)	52.42 (1.46)	0.80
Weight kg Mean (SD)	72.12 (14.95)	68.50 (13.27)	< 0.001
*Socio-demographic characteristics*			
Education (%)			
No post high school	16.3	17.9	0.62
Trade/Diploma	21.5	21.5	
University or higher	62.2	60.6	
Marital status (%)			
Married/de facto	80.2	84.1	0.05
Separated/divorced/widowed	16.3	13.5	
Single	3.5	2.4	
Area (%)			
Urban	42.6	38.5	0.13
Rural	52.7	56.1	
Remote	4.3	5.5	
*Lifestyle characteristics*			
Sitting time hours per day (mean (SD))	5.83 (3.11)	5.76 (3.04)	0.59
Physical activity MET.mins (median (IQR))	540 (135–1200)	540 (180–1215)	0.21
BMI (%)			
Underweight	1.2	1.8	< 0.001
Healthy weight	39.9	50.2	
Overweight	35.5	31.0	
Obese	24.4	17.0	
Smoking (%)			
Current	13.9	12.2	0.04
Ex-smoker	25.4	21.8	
Never smoked	60.7	66.0	
Alcohol (%)			
Risky	6.0	5.2	0.75
Non-risky	82.6	83.6	
Never drink	11.4	11.2	
*Health characteristics*			
Chronic disease (median (IQR))^c^	0 (0–1)	0 (0–1)	< 0.001
Depressive symptoms (median (IQR))	5 (3–9)	4 (2–7)	< 0.001
Pain (%)			
Low	56.2	74.0	< 0.001
Medium	26.6	17.4	
Severe	17.2	8.6	
Physical function (%)			
Low	14.8	7.0	< 0.001
Medium	33.4	24.8	
High	51.8	68.3	
Menopausal status (%)			
Surgical menopause	32.0	22.3	< 0.001
HRT use	16.6	15.6	
OCP use	4.4	2.5	
Pre-menopausal	9.3	11.2	
Peri-menopausal	17.7	20.9	
Post-menopausal	20.0	27.5	

^a^Baseline for the analyses in this paper.

^b^Chi-square for categorical and t-tests for continuous variables.

^c^From a list including: diabetes, heart disease, hypertension, stroke, asthma, chronic bronchitis or emphysema, osteoporosis, breast, cervical and other cancers.

BMI was based on self-reported weight (kg) and height (m), calculated and categorized following WHO guidelines^19^. Smoking status was based on responses to questions about current and past use of cigarettes or other tobacco products, and categorised as ‘current’, ‘ex-smoker’ or ‘never smoked’^20^. Alcohol consumption was based on responses to questions about frequency, number of drinks, and how often five or more drinks were consumed on one occasion. Risk categories were based on National Health and Medical Research Council (NHRMC) guidelines and categorised as ‘risky’, ‘non-risky’ or ‘never drink’^21^.

Chronic disease was based on the number of self-reported doctor diagnosed conditions, from a list which included diabetes; heart disease; hypertension; stroke; asthma, chronic bronchitis or emphysema; osteoporosis; breast, cervical or other cancers. Depressive symptoms were assessed using the 10-item Center for Epidemiologic Studies Depression Scale^22^. Scores range from 0 to 30 with higher scores indicting more depressive symptoms. Pain and physical function were assessed using subscales of the SF-36^23^. The score for both scales ranges from 0 to 100 with higher scores indicating lower pain and better physical functioning. Scores were categorised as follows: for pain, < 50 = ‘severe’, 50 < 70 = ‘medium’, ≥ 70 = ‘low’; for physical function, < 70 = ‘low’, 70 < 90 = ‘medium’, ≥ 90 = ‘high’^24,25^. Menopausal status was categorised as ‘surgical menopause’, ‘HRT use’, ‘OCP use’, ‘pre-’, ‘peri-’, or ‘post-menopausal’^26^.

Positive lifestyle changes were defined as self-reported weight loss of ≥ 5 kg, a reduction in sitting time > 1 h/day, an increase in physical activity (PA) > 150 metabolic equivalent (MET).min/week (equivalent to 45 min/week of moderate intensity activity^27^, smoking cessation, and a reduction in alcohol consumption (from non-risky to risky or never drink) and smoking cessation. Negative lifestyle changes were defined as weight gain of ≥ 5 kg, an increase in sitting time > 1 h/day, a decrease in PA > 150 MET.min/week, starting smoking (never or ex-smoker to current smoker), and an increase in alcohol consumption (from non-risky/never drink to risky).

### Statistical analyses

Descriptive statistics were used to summarize the socio-demographic and health-related characteristics of the No OA and OA groups. Women with missing data for lifestyle or health characteristics (N = 299, 6.3%) were excluded from the analyses. Socio-demographic and health-related characteristics of women with missing data and complete data are presented in Supplementary Table 1A.

For each lifestyle factor, chi-square tests were used to examine the differences between the No OA and OA groups in the proportions of women, who showed (a) a positive change in behaviour, and (b) a negative change in behaviour. We used logistic regression analyses to examine the odds of adopting positive and negative changes in behaviour in the OA and No OA groups. These comparisons were made for three survey intervals: i.e., from 2001 to 2004, reflecting the period of time prior to diagnosis; from 2004 to 2007, reflecting the period of time around diagnosis; and from 2007 to 2010, reflecting the period of time following diagnosis. The differences in sample sizes for each behaviour change, as indicated in Tables 2 and 3, are due to the different inclusion criteria for each behaviour, depending on who was able to improve behaviour (i.e., those who already demonstrated healthy behaviour were excluded) or whose behaviour could deteriorate (i.e., those who already demonstrated unhealthy behaviour were excluded). To examine whether the pattern of lifestyle change differed in the OA and No OA groups over time, we used generalizing estimating equations with interaction terms for OA and survey interval. To examine the potential role of joint pain and stiffness in the lifestyle changes, we conducted post-hoc analyses. Within the OA group we examined the association between frequency of joint pain and stiffness and positive and negative lifestyle changes using logistic regression. Statistical analyses were conducted in SPSS version 28 (IBM Corporation, NY, USA) with significance level set at p < 0.05 and confidence interval at 95% CI.

**Table 2:** Positive lifestyle changes in 2001–2004 (prior to diagnosis), 2004–2007 (around diagnosis) and 2007–2010 (following diagnosis)-in women who were and were not diagnosed with OA between 2004 and 2007.

Lifestyle change	Women diagnosed with OA		Women not diagnosed with OA		p-value	OR (95% CI)^c^	p-value for interaction of OA with time
	*N*	% who made a positive change	*N*	% who made a positive change			
Weight loss (≥ 5 kg)							
Period prior to OA diagnosis (2001–04)	517	10.1	3342	10.1	0.95	0.98 (0.92, 1.04)	0.04
Period around OA diagnosis (2004–07)	538	13.0	3452	11.4	0.26	1.00 (0.94, 1.06)	
Period following OA diagnosis (2007–10)	564	14.4	3700	9.8	< 0.001	1.05 (0.99, 1.10)	
Total sitting time (reduce > 1 h/day)							
Period prior to OA diagnosis	523	34.6	3266	24.9	< 0.001	1.09 (1.05, 1.14)	0.61
Period around OA diagnosis	467	36.4	3209	24.5	< 0.001	1.11 (1.06, 1.16)	
Period following OA diagnosis	542	39.1	3386	29.5	< 0.001	1.07 (1.03, 1.11)	
Increase in PA (increase > 150 MET.min/week)							
Period prior to OA diagnosis	518	50.8	3406	48.1	0.25	1.02 (0.98, 1.06)	0.22
Period around OA diagnosis	510	40.8	3347	43.8	0.20	0.98 (0.94, 1.02)	
Period following OA diagnosis	522	38.5	3479	40.3	0.43	0.99 (0.95, 1.03)	
Stopped smoking^a^							
Period prior to OA diagnosis	85	78.8	435	16.1	< 0.001	1.86 (1.64, 2.10)	0.32
Period around OA diagnosis	73	83.6	406	26.4	< 0.001	1.72 (1.53, 1.94)	
Period following OA diagnosis	70	88.6	335	17.9	< 0.001	1.81 (1.60, 2.05)	
Reduction in alcohol^b^							
Period prior to OA diagnosis	32	28.1	181	30.4	0.80	0.98 (0.82, 1.17)	0.37
Period around OA diagnosis	34	38.2	261	41.0	0.76	0.97 (0.83, 1.14)	
Period following OA diagnosis	36	22.2	258	36.4	0.09	0.86 (0.72, 1.02)	

^a^Excludes all ex- and non-smokers.

^b^Excludes all non-risky alcohol drinkers.

^c^Odds ratio adjusted for chronic diseases, depressive symptoms, pain, physical function, menopausal status, OR (95% CI) is the odds in the OA group versus the No-OA group.

**Table 3:** Negative lifestyle changes in 2001–2004 (prior to diagnosis), 2004–2007 (around diagnosis) and 2007–2010 (following diagnosis)-in women who were and were not diagnosed with OA between 2004 and 2007.

Lifestyle change	Women diagnosed with OA		Women not diagnosed with OA		p-value	OR (95% CI)^c^	p-value for interaction of OA with time
	N	% who made a negative change	N	% who made a negative change			
Weight gain (≥ 5 kg)							
Period prior to OA diagnosis (2001–04)	517	20.9	3342	18.0	0.12	1.02 (0.97, 1.07)	0.22
Period around OA diagnosis (2004–07)	538	21.4	3452	14.5	< 0.001	1.08 (1.03, 1.14)	
Period following OA diagnosis (2007–10)	564	16.8	3700	16.8	0.96	0.97 (0.92, 1.02)	
Total sitting time (increase > 1 h/day)							
Period prior to OA diagnosis	523	41.3	3266	36.4	0.03	1.04 (1.00, 1.08)	0.57
Period around OA diagnosis	467	38.3	3209	35.1	0.18	1.02 (0.98, 1.07)	
Period following OA diagnosis	542	38.4	3386	32.3	0.005	1.07 (1.03, 1.11)	
Decrease in PA (decrease > 150 MET.mins/week)							
Period prior to OA diagnosis	518	29.2	3406	30.6	0.50	1.00 (0.96, 1.05)	0.58
Period around OA diagnosis	510	37.5	3347	36.6	0.71	1.02 (0.98, 1.06)	
Period following OA diagnosis	522	38.9	3479	39.6	0.76	1.02 (0.98, 1.07)	
Started smoking^a^							
Period prior to OA diagnosis	519	10.8	3229	1.3	< 0.001	1.56 (1.43, 1.70)	0.30
Period around OA diagnosis	495	9.3	3247	0.7	< 0.001	1.69 (1.52, 1.88)	
Period following OA diagnosis	559	8.9	3490	0.8	< 0.001	1.66 (1.49, 1.84)	
Increase in alcohol^b^							
Period prior to OA diagnosis	503	1.6	3293	3.7	0.02	0.82 (0.71, 0.95)	0.33
Period around OA diagnosis	533	2.4	3367	2.1	0.59	1.04 (0.92, 1.17)	
Period following OA diagnosis	541	1.5	3551	2.3	0.21	0.93 (0.79, 1.08)	

^a^Includes all ex- and non-smokers.

^b^Includes all non-risky alcohol drinkers.

^c^Odds ratio adjusted for chronic diseases, depressive symptoms, pain, physical function, menopausal status, OR (95% CI) is the odds in the OA group versus the No-OA group.

## Results

The socio-demographic and health-related characteristics of the sample of 610 women with OA diagnosed between 2004 and 2007, and the 3810 women who never reported OA, are shown in Table 1. In 2001, the mean age of both groups was 52 (± 1.5) years. Women with OA had significantly higher weight and BMI than women without OA (p < 0.001). Sitting time and physical activity were similar in both groups. Overall, the OA group tended to be less healthy, they reported more chronic diseases, depression and pain, and lower physical function, and were more likely to have had surgical menopause than the No OA group. There were no differences in the socio-demographic characteristics of the two groups. Comparison of women with complete data and women with missing data on any of the confounding variables, showed that there were some statistically significant differences (Supplementary Table 1A). However, the absolute differences between the groups were small.

In the period following diagnosis (2007–2010), 38% of women with OA adopted one positive lifestyle change and a further 15% adopted at least two positive lifestyle changes. Corresponding estimates for the No OA group were 28% (one) and 6% (two). Compared with the No OA group, women in the OA group were more likely to adopt positive lifestyle changes (Table 2). A greater proportion of women with OA lost ≥ 5 kg in the period following OA diagnosis (14.4% vs. 9.8%, p < 0.001). Across all three survey intervals, more women with OA (than women without OA) reduced sitting time by at least an hour/day (odds ratio [OR]s = 1.07–1.11, p < 0.001). Moreover, more than three quarters of the women with OA who were smokers, stopped smoking, compared with a up to a quarter of the women who were smokers in the No OA group (ORs = 1.72–1.86, p < 0.001). No statistically significant differences were found for positive changes in physical activity and alcohol intake. The only significant interaction term for positive lifestyle changes and time was for weight loss (p = 0.04), indicating that the 9 year trend in weight loss was more favourable in the OA group than in the non OA group.

In the OA group, 35% of women adopted one negative lifestyle change and a further 6% adopted at least two negative lifestyle changes. Corresponding estimates in the No OA group were 29% and 5%. Compared with the No OA group, women in the No OA group were more likely to adopt negative lifestyle changes (Table 3). More women with OA gained ≥ 5 kg around the OA diagnosis, (21.4% vs. 14.5%, p < 0.001, OR = 1.08, confidence interval [CI] = 1.03; 1.14). Proportionally, more women with OA than without OA increased their sitting by at least one hour prior to (p = 0.03, OR = 1.04, CI = 1.00; 1.08) and following the OA diagnosis (p = 0.005, OR = 1.07, CI = 1.03; 1.11). At all three survey intervals, a greater proportion of women with OA than without OA, who had never smoked or were ex-smokers, started smoking (ORs = 1.56–1.69, p < 0.001). Very few women reported increases in alcohol intake, but the proportion who did this prior to diagnosis was higher in the No OA group than the OA group (p = 0.02, OR = 0.82 CI = 0.71; 0.95). There were no significant interaction terms for negative lifestyle changes and time.

In the post-hoc analyses of data from women with OA, those who reported ’often’ having joint pain and stiffness were more likely to gain more than 5 kg following OA diagnosis than those who reported pain ‘never’, ‘rarely’ or ‘sometimes’ (p = 0.01, OR = 1.63, CI = 1.03; 2.58) (Supplementary Table 3A). There were no other significant differences between groups for making positive or negative lifestyle changes (Supplementary Table 2A, 3A).

## Discussion

This study describes changes in lifestyle behaviours in women who did/did not report being diagnosed with OA for the first time in 2007. Over a 9 year period, more women with OA reported positive changes in weight (lost ≥ 5 kg), sitting time (reduced by > 1 h/day) and smoking (quit) in the three year period following OA diagnosis, than women without OA in the same three year period. There were however some negative changes in these behaviours. For example, many more women with OA (than without OA) reported starting smoking in the period following OA diagnosis.

Our findings are encouraging, because they show that some women who are diagnosed with OA make changes in line with those suggested in the clinical guidelines for non-surgical management of hip and knee OA, established by The Royal Australian College of General Practitioners (RACGP), the OA Research Society International (OARSI) and the European League Against Rheumatism (EULAR)^28–32^. The strongest evidence based non-pharmacological management recommendations include weight reduction and land based exercise^45^. In our study, approximately 67% of the women with OA were overweight or obese, and of these, about one in six lost weight following OA diagnosis (compared with one in ten in the No OA group). This is important, because obesity is a major risk factor for the onset and progression of OA^33^. and weight gain is associated with an increase in arthritis symptoms, while weight loss is associated with a reduction in symptoms^34^. It is important that practitioners continue to encourage overweight patients with OA to lose 10% or more of body weight, as there is a dose–response relationship between weight loss and pain and functional status^34^.

Most OA guidelines recognise the validity, safety and cost effectiveness of exercise over pharmacological therapies and recommend that promoting PA according to public health recommendations be part of standard care in people with OA^35,36^. Established recommendations suggest PA is an important modifiable management strategy that can reduce pain and improve physical function in people living with OA^29–32^. Evidence suggests that 13–60% of people with knee and hip OA meet recommended levels of PA^37^, and that people with lower limb OA are challenged to participate in PA due to increased symptoms, such as impaired physical function and weight bearing pain^38^. In our study, more than half the women in both the OA and non-OA groups met current PA guidelines, as evidenced by the median PA score in both groups of 540 MET.mins/week., which is slightly above the 500 MET.min/week threshold^39^. Although approximately 40% of women increased PA during and after diagnosis, a similar proportion reduced their PA, with similar findings in the No OA group. This suggests that the PA changes were not related to diagnosis of OA. This is in line with findings from previous studies of the same cohort, which showed that diagnosis with a chronic condition was not associated with changes in PA^40^. Where symptoms permit, women with OA should consider meeting guidelines for physical activity and sedentary behaviour by accumulating activities which amount to at least 500 MET.minutes/week (which is at least 150–300 min of moderate-intensity, or at least 75–150 min of vigorous-intensity aerobic physical activity, or an equivalent combination through the week). Both the PA and OA guidelines also suggest that people with OA should also do muscle-strengthening activities at least twice a week.

PA guidelines also suggest that adults should reduce/break-up long periods of sedentary time^27^ and there is emerging evidence that high levels of sedentary behaviour are associated with poorer physical function in people with knee OA^41^. A study utilising accelerometers has shown that people with knee OA spent 60% of waking time in sedentary behaviour^42^. Our findings indicate that more women with OA were more likely to reduce their sitting time by at least an hour per day in each of the survey periods, than women without OA. In contrast, compared with women without OA, slightly more women who were diagnosed with OA reported increasing sitting time by an hour following diagnosis. It is unclear if these changes in sitting time were due to efforts in managing OA symptoms, or to growing awareness of the adverse health effects of too much sitting^39^. In another study from the same cohort, several life events were associated with either decreasing or increasing sitting time; retirement and changes at work were important predictors of change^43^. A recent study has reported that women with 8–10 h of sedentary behaviour per day are 1.37 times more likely to suffer from chronic knee pain than those who sit < 5 h per day^44^.

Associations between smoking and OA are inconsistent. In the Framingham OA Study, smoking was protective against development of knee OA^45^, but the mechanisms underlying this effect remain unclear. In another cohort study of 2505 men and women aged 40 years and older, there was no association between smoking and the development of OA^46^. In the current study, a significantly large proportion (~ 80%) of the women with OA who were smokers quit smoking across the three survey intervals. However, a small but significant proportion of non-smoking women with OA (~ 10%) also started smoking. It is unclear if these changes in smoking status were related to diagnosis of OA, but is possible that some women started smoking to help control their weight^47^. Given the obvious negative health impacts of smoking, we do not advocate smoking to assist with weight control, but instead suggest that the effects of nicotine as a therapeutic agent for managing pain in OA, should be investigated^48^.

The evidence that alcohol consumption is associated with OA is limited, but evidence suggests protective effects on developing rheumatoid arthritis^49^. One cohort study has reported that consuming > 3 glasses of alcohol per week over 10 years was associated with a 52% decreased risk of rheumatoid arthritis^50^. In the current study, more women increased their alcohol consumption before diagnosis of OA (than women in the No OA group), but the prevalence was very low. As with smoking, the association between alcohol consumption and the development and exacerbation of OA remains unclear, but practitioners should acknowledge the negative health impact of excess alcohol consumption when considering treatments for OA patients and recommend meeting guidelines for alcohol consumption^51^.

The post-hoc analyses showed no strong evidence that the likelihood of adopting positive or negative lifestyle changes was driven by the frequency of experiencing joint symptoms (Supplementary Tables 2A and 3A). However, the numbers of participants who made changes in some of the categories were small, resulting in wide confidence intervals and low statistical power to detect statistically significant associations. Hence, verification of the findings in a different sample is recommended. While lifestyle changes are recommended, self-management of chronic pain also involves assessing and adapting to ‘risks’ of pain, the environment, and continuity with valued and daily activities^52^. Lived experiences of illness and pain in relation to socially situated physical environments may explain the varying adoption of lifestyle changes.

The strengths of this study include the large sample size, nine year follow-up and the range of lifestyle factors measured, which allowed examination of changes in lifestyle factors over a long period of time. Given that OA is reported by twice as many women as men, and that more women engage in health-promoting behaviours, this study improves our understanding of how women do and do not respond to current guidance on management of OA. To our knowledge this is the first time women’s behaviours around the time of OA diagnosis have been reported. There are however several limitations. First, although the study relied on self-reported behaviours almost all the ALSWH questions have acceptable reliability and validity^26^. Second, the data do not specify which joint is affected, and lifestyle changes may be more important for women with knee and hip OA (than for those with other forms of OA), because these forms of OA are associated with obesity^34^. Due to the self-reported nature of the data, it is possible that there may have been some misclassification of OA, and a small proportion of women may have been unaware of other forms of arthritis. However, others have shown it is justifiable to interpret participants who indicate ‘other arthritis’ as having OA^53^. Third, while a benefit of the observational nature of this study is that we were able to describe the natural behaviours of participants, we cannot imply any causal relationships between OA diagnosis and lifestyle changes. Participants may have had other motivations to change behaviour than OA, such as other life events and the onset of other diseases and comorbidities, or prevailing health promotion campaigns^54^. Fourth, data from 6.3% of the women who met inclusion criteria could not be included in the analyses due to missing values. This could potentially have led to selection bias. However, comparison of women with complete data and those with missing data showed only minor differences between the groups, suggesting limited selection bias. Finally, the over-representation of Australia-born and university-educated women in the sample means that the results may not be applicable to all Australian women.

In conclusion, this study found that more than one third of women who were diagnosed with OA made positive lifestyle changes, at the time of, or following, diagnosis with OA. However another third also made negative lifestyle changes, especially relating to smoking. As diagnosis with OA appears to be a time when lifestyle change is possible, the challenge is to develop strategies that will encourage more women who are diagnosed with OA to make more positive lifestyle changes. A comprehensive management plan is vital for patients’ self-management of the disease, which can lead to effective adoption of lifestyle strategies and sustained benefits. It is vital for primary care practitioners to not only provide newly diagnosed patients with information and advice about the disease, but to also provide advice and support for increasing PA, reducing sedentary behaviour, and dietary change, to achieve weight loss and maintenance of healthy weight, as a first-line intervention in managing the symptoms of OA.

### Supplementary Information

Supplementary Information.

## Data Availability

The data that support the findings of this study are available from the ALSWH but restrictions apply to the availability of these data, which were used under license for the current study, and so are not publicly available. Data are however available from the authors upon reasonable request and with permission of ALSWH Data Access Committee. ALSWH survey data are owned by the Australian Government Department of Health and due to the personal nature of the data collected, release by ALSWH is subject to strict contractual and ethical restrictions. Ethical review of ALSWH is by the Human Research Ethics Committees at The University of Queensland and The University of Newcastle. De-identified data are available to collaborating researchers where a formal request to make use of the material has been approved by the ALSWH Data Access Committee. The committee is receptive of requests for datasets required to replicate results. Information on applying for ALSWH data is available from https://alswh.org.au/for-data-users/applying-for-data/.
