# Supplementary material for: Lifestyle behaviour changes associated with osteoarthritis: a prospective cohort study
Source: Sci Rep. 2024 Mar 14;14:6242. doi: 10.1038/s41598-024-54810-6 (PMC10940587; doi:10.1038/s41598-024-54810-6)
Supplement: Supplementary file 1 — Supplementary Information. [file 41598_2024_54810_MOESM1_ESM.docx]

**Supplementary Table 1A:** Socio-demographic, lifestyle and health-related characteristics of women with missing data and women with complete data between 2004 and 2007. All data are from the 2001 survey^a^ (N = 4719)

|  | Women with missing data (n=299) | Women with complete data (n=4420) | p-value^b^ |
| --- | --- | --- | --- |
| Age yrs Mean (SD) | 52.62 (1.45) | 52.42 (1.46) | 0.03 |
| Weight kg Mean (SD) | 68.85 (13.27) | 69.02 (13.59) | 0.84 |
| **Socio-demographic characteristics** |  |  |  |
| Education (%) |  |  |  |
| No post high school | 13.8 | 17.7 | 0.15 |
| Trade/Diploma | 20.2 | 21.5 |  |
| University or higher | 66.0 | 60.8 |  |
| Marital status (%) |  |  |  |
| Married/de facto | 82.4 | 83.6 | 0.09 |
| Separated/divorced/widowed | 12.8 | 13.9 |  |
| Single | 4.8 | 2.6 |  |
| Area (%) |  |  |  |
| Urban | 34.2 | 93.0 | 0.24 |
| Rural | 59.4 | 55.6 |  |
| Remote | 6.5 | 5.3 |  |
| **Lifestyle characteristics** |  |  |  |
| Sitting time hours per day (mean (SD)) | 5.32 (3.22) | 5.80 (3.04) | 0.02 |
| Physical activity MET.mins (median (IQR)) | 530 (90-1178) | 540 (180-1200) | 0.16 |
| BMI (%) |  |  |  |
| Underweight | 1.1 | 1.7 | 0.31 |
| Healthy weight | 45.1 | 48.8 |  |
| Overweight | 36.8 | 31.5 |  |
| Obese | 16.9 | 18.0 |  |
| Smoking (%) |  |  |  |
| Current | 9.3 | 12.4 | 0.21 |
| Ex-smoker | 25.1 | 22.3 |  |
| Never smoked | 65.6 | 65.3 |  |
| Alcohol (%) |  |  |  |
| Risky | 3.6 | 5.3 | 0.03 |
| Non-risky | 80.4 | 83.5 |  |
| Never drink | 15.9 | 11.2 |  |
|  |  |  |  |
| **Health characteristics** |  |  |  |
| Chronic disease (median (IQR))^c^ | 0 (0-1) | 0 (0-1) | 0.12 |
| Depressive symptoms (median (IQR)) | 5 (2-9) | 4 (2-7) | 0.04 |
| Pain (%) |  |  |  |
| Low | 66.6 | 71.5 | 0.07 |
| Medium | 19.7 | 18.7 |  |
| Severe | 13.8 | 9.8 |  |
| Physical function (%) |  |  |  |
| Low | 12.7 | 8.1 | 0.03 |
| Medium | 25.4 | 26.0 |  |
| High | 61.9 | 66.0 |  |
| Menopausal status (%) |  |  |  |
| Surgical menopause | 26.4 | 23.6 | 0.18 |
| HRT use | 19.8 | 15.8 |  |
| OCP use | 1.2 | 2.8 |  |
| Pre-menopausal | 9.3 | 11.0 |  |
| Peri-menopausal | 17.1 | 20.5 |  |
| Post-menopausal | 26.4 | 26.5 |  |
| ^a^ Baseline for the analyses in this paper  ^b^ Chi-square for categorical and t-tests for continuous variables  ^c^  From a list including: diabetes, heart disease, hypertension, stroke, asthma, chronic bronchitis or emphysema, osteoporosis, breast, cervical and other cancers | | | |

**Supplementary Table 2A:** Positive lifestyle changes in women who were diagnosed with OA: comparison of women reporting joint pain and stiffness often versus women reporting joint pain and stiffness never, rarely or sometimes.

|  | Joint pain and stiffness (never, rarely, sometimes) | |  | Joint pain and stiffness (often) | |  | |  |  |
| --- | --- | --- | --- | --- | --- | --- | --- | --- | --- |
| Lifestyle change | N | % who made a positive change | | N | % who made a positive change | | p-value | | OR (95%CI) |
| Weight loss (≥ 5 kg) |  |  | |  |  | |  | |  |
| Period prior to OA diagnosis (2001-04) | 328 | 8.5 | | 56 | 10.7 | | 0.60 | | 1.44 (0.51, 4.04) |
| Period around OA diagnosis (2004-07) | 422 | 11.9 | | 94 | 19.2 | | 0.06 | | 1.50 (0.77, 2.93) |
| Period following OA diagnosis (2007-10) | 339 | 15.9 | | 217 | 11.5 | | 0.15 | | 0.63 (0.37, 1.07) |
| Total sitting time (reduce > 1hr/day) |  |  | |  |  | |  | |  |
| Period prior to OA diagnosis | 327 | 33.6 | | 53 | 32.1 | | 0.82 | | 0.79 (0.39, 1.59) |
| Period around OA diagnosis | 365 | 35.1 | | 81 | 43.2 | | 0.17 | | 1.28 (0.75, 2.18) |
| Period following OA diagnosis | 312 | 42.6 | | 221 | 34.8 | | 0.07 | | 0.74 (0.51, 1.08) |
| Increase in PA (increase > 150 MET.min/week) |  |  | |  |  | |  | |  |
| Period prior to OA diagnosis | 330 | 53.6 | | 54 | 40.7 | | 0.08 | | 0.64 (0.33, 1.23) |
| Period around OA diagnosis | 400 | 41.5 | | 89 | 39.33 | | 0.71 | | 0.90 (0.53, 1.52) |
| Period following OA diagnosis | 315 | 37.5 | | 200 | 40.5 | | 0.49 | | 1.13 (0.78, 1.64) |
| Stopped smoking ^a^ |  |  | |  |  | |  | |  |
| Period prior to OA diagnosis | 41 | 82.9 | | 16 | 68.8 | | 0.24 | | 0.22 (0.02, 2.44) |
| Period around OA diagnosis | 53 | 86.8 | | 15 | 80.0 | | 0.51 | | 0.25 (0.03, 2.31) |
| Period following OA diagnosis | 43 | 90.7 | | 25 | 84.0 | | 0.41 | | 0.49 (0.08, 2.93) |
| Reduction in alcohol ^b^ |  |  | |  |  | |  | |  |
| Period prior to OA diagnosis | 15 | 33.3 | | 5 | 20.0 | | 0.57 | | n.a. |
| Period around OA diagnosis | 29 | 37.9 | | 4 | 50.0 | | 0.64 | | 1.09 (0.03, 36.9) |
| Period following OA diagnosis | 24 | 25.0 | | 12 | 16.7 | | 0.57 | | 2.41 (0.05, 128.3) |

^a^ excludes all ex- and non-smokers

^b^ excludes all non-risky alcohol drinkers

n.a. not a number; odds ratio and confidence interval could not be determined due to few cases per category.

**Supplementary Table 3A:** Negative lifestyle changes in women who were diagnosed with OA: comparison of women reporting joint pain and stiffness often versus women reporting joint pain and stiffness never, rarely or sometimes.

|  | Joint pain and stiffness (never, rarely, sometimes) | |  | Joint pain and stiffness (often) | |  |  | |  |
| --- | --- | --- | --- | --- | --- | --- | --- | --- | --- |
| Lifestyle change | N | % who made a negative change | | N | % who made a negative change | p-value | | OR (95%CI) | |
| Weight gain (≥ 5 kg) |  |  | |  |  |  | |  | |
| Period prior to OA diagnosis (2001-04) | 328 | 21.0 | | 56 | 23.2 | 0.71 | | 1.09 (0.51, 2.33) | |
| Period around OA diagnosis (2004-07) | 422 | 18.5 | | 94 | 26.6 | 0.08 | | 1.58 (0.86, 2.88) | |
| Period following OA diagnosis (2007-10) | 339 | 13.9 | | 217 | 22.1 | 0.01 | | 1.63 (1.03, 2.58) | |
| Total sitting time (increase > 1hr) |  |  | |  |  |  | |  | |
| Period prior to OA diagnosis | 327 | 39.8 | | 53 | 45.3 | 0.45 | | 1.41 (0.72, 2.74) | |
| Period around OA diagnosis | 365 | 40.8 | | 81 | 29.6 | 0.06 | | 0.61 (0.35, 1.07) | |
| Period following OA diagnosis | 312 | 36.9 | | 221 | 40.3 | 0.43 | | 1.14 (0.79, 1.64) | |
| Decrease in PA (decrease > 150 MET.mins/week) |  |  | |  |  |  | |  | |
| Period prior to OA diagnosis | 330 | 27.9 | | 54 | 33.3 | 0.41 | | 1.37 (0.68, 2.77) | |
| Period around OA diagnosis | 400 | 37.8 | | 89 | 32.6 | 0.36 | | 0.90 (0.53, 1.54) | |
| Period following OA diagnosis | 315 | 41.6 | | 200 | 35.0 | 0.14 | | 0.81 (0.55, 1.19) | |
| Started smoking ^a^ |  |  | |  |  |  | |  | |
| Period prior to OA diagnosis | 336 | 11.6 | | 50 | 12.0 | 0.94 | | 1.21 (0.44, 3.32) | |
| Period around OA diagnosis | 391 | 9.2 | | 85 | 10.6 | 0.69 | | 1.41 (0.60, 3.33) | |
| Period following OA diagnosis | 332 | 9.3 | | 219 | 7.8 | 0.52 | | 0.73 (0.38, 1.39) | |
| Increase in alcohol ^b^ |  |  | |  |  |  | |  | |
| Period prior to OA diagnosis | 324 | 1.9 | | 55 | 1.8 | 0.99 | | 0.82 (0.06, 11.47) | |
| Period around OA diagnosis | 415 | 2.4 | | 95 | 1.1 | 0.41 | | 0.32 (0.03, 3.22) | |
| Period following OA diagnosis | 323 | 0.9 | | 210 | 2.4 | 0.18 | | 2.63 (0.58, 12.05) | |

^a^ includes all ex- and non-smokers

^b^ includes all non-risky alcohol drinkers
